# Evaluation of toxoplasmosis in pregnant women using dot-immunogold-silver staining with recombinant *Toxoplasma gondii* peroxiredoxin protein

**DOI:** 10.1186/s12879-020-05414-8

**Published:** 2020-09-22

**Authors:** Zhuan-zhuan Liu, Xue-yan Li, Lin-lin Fu, Fei Yuan, Ren-xian Tang, Yi-sheng Liu, Kui-yang Zheng

**Affiliations:** Jiangsu Key Laboratory of Immunity and Metabolism, Department of Pathogen Biology and Immunology, Xuzhou Medical University, Xuzhou, 221004 China

**Keywords:** *Toxoplasma gondii*, Peroxiredoxin, Dot-IGSS, Serum, Pregnant women

## Abstract

**Background:**

*Toxoplasma gondii* infection endangers human health and affects animal husbandry. Serological detection is the main method used for epidemiological investigations and diagnosis of toxoplasmosis. The key to effective diagnosis of toxoplasmosis is the use of a standardized antigen and a specific and sensitive detection method. Peroxiredoxin is an antigenic protein and vaccine candidate antigen of *T. gondii* that has not yet been exploited for diagnostic application.

**Methods:**

In this study, recombinant *T. gondii* peroxiredoxin protein (rTgPrx) was prepared and used in dot-immunogold-silver staining (Dot-IGSS) to detect IgG antibodies in serum from mice and pregnant women. The rTgPrx-Dot-IGSS method was established and optimized using mouse serum. Furthermore, serum samples from pregnant women were analyzed by rTgPrx-Dot-IGSS.

**Results:**

Forty serum samples from mice infected with *T. gondii* and twenty negative serum samples were analyzed. The sensitivity and specificity of rTgPrx-Dot-IGSS were 97.5 and 100%, respectively, equivalent to those of a commercial ELISA kit for anti-*Toxoplasma* IgG antibody. Furthermore, 540 serum samples from pregnant women were screened with a commercial ELISA kit. Eighty-three positive and 60 negative serum samples were analyzed by rTgPrx-Dot-IGSS. The positive rate was 95.18%, comparable to that obtained with the commercial ELISA kit.

**Conclusions:**

The Dot-IGSS method with rTgPrx as an antigen might be useful for diagnosing *T. gondii* infection in individuals.

## Background

*Toxoplasma gondii*, the causative agent of zoonotic toxoplasmosis, threatens the health of 30% ~ 50% of the population worldwide [1]. Most infections are asymptomatic, but toxoplasmosis can cause abortion, stillbirth, and severe congenital toxoplasmosis in pregnant women and life-threatening *Toxoplasma* encephalitis in immunocompromised patients, such as those with HIV and those who have undergone organ transplantation [2]. Between January 1988 and December 2018, the global prevalence of acute toxoplasmosis in pregnant women was 1.1%; the highest prevalence was in the Eastern Mediterranean region, and the lowest was in Europe [3]. An estimated 190,100 cases of congenital toxoplasmosis are diagnosed annually worldwide [4, 5]. In China, the seroprevalence of *T. gondii* in pregnant women ranges from 2.4 to 5.0% and is as high as 16.29% in pregnant Manchu women [6, 7]. Because the optimal treatment strategy for toxoplasmosis is unclear, early diagnosis and intervention are very important so that it can be prevented [8].

Many stages of *T. gondii* can exist in different anatomical locations; thus, diagnosis by etiological methods is difficult. Serological testing is the most commonly used method for clinical diagnosis of *T. gondii* infection [9]. Enzyme-linked immunosorbent assay (ELISA) is often applied to detect antibodies (IgG, IgM, IgA and IgE) in serum [9, 10]. This simple method can be used to test many samples simultaneously [11]. However, the quality of commercially available *T. gondii* detection kits is inconsistent, and information on specificity and sensitivity is often lacking [6]. Sensitive, specific and rapid immunological detection methods for toxoplasmosis have long been explored and are greatly needed.

The dot-immunogold-silver staining (Dot-IGSS) method uses the specificity of antigen-antibody binding and the sensitivity of gold-silver particles to detect serum antibodies in patients with parasitic diseases [12, 13]. The sensitivity of Dot-IGSS are higher than those of ELISA for diagnosing schistosomiasis, clonorchiasis, toxoplasmosis and cysticercosis [13–16]. The Dot-IGSS procedure is simple and convenient and, unlike ELISA, does not require a microplate reader. Therefore, Dot-IGSS can be carried out in township hospitals and community health service centers.

Antigen is a key element in diagnostic methods. Soluble tachyzoite antigen (STAg) and excretory secretion antigen (ESA) of *T. gondii* are common diagnostic antigens, but these antigens exhibit specificity for certain species and *T. gondii* strains and are thus difficult to standardize [9, 11]. *T. gondii* peroxiredoxin protein (TgPrx) is an antigenic protein in STAg that has been demonstrated to be detectable by 2-dimensional electrophoresis (2-DE), mass spectrometry (MS) and Western blotting with rabbit anti-*T. gondii* serum [17]. Recombinant TgPrx (rTgPrx) can induce humoral and cellular immune responses that protect mice against lethal *T. gondii* infection [18]. rTgPrx is thus a novel vaccine antigen for toxoplasmosis, but little is known about its diagnostic applications.

Here, rTgPrx was prepared, purified and used as a standardized antigen. We then combined the sensitivity of Dot-IGGS with the specificity of rTgPrx to detect antibodies against *T. gondii* in serum, demonstrating a new and convenient diagnostic method for toxoplasmosis.

## Methods

### Ethics statement

The animal model was established according to the Guidelines for the Laboratory Animal Use and Care Committee of the Ministry of Health, China, and the Ethics Committee on Animal Research of Xuzhou Medical University (No. SCXK < SU > 2014–0003). All serum samples from pregnant women were provided by Xuzhou Maternity and Child Health Care Hospital. Written informed consent was provided by each participant.

### Preparation of rTgPrx

The recombinant plasmid pGEX-6P-1/TgPrx was constructed. The positive plasmid was transformed into *E. coli* BL21 and induced by isopropyl-β-D-thiogalactoside (IPTG) [19]. Soluble rTgPrx was purified via glutathione S-transferase (GST) affinity chromatography and identified by Western blotting. PreScission Protease (GE Healthcare, U.S.A.) was used to cleave the GST tag from the rTgPrx fusion protein. The purity of rTgPrx was calculated using Imag J software [20]. The concentration of rTgPrx was measured using a BCA protein assay kit (Thermo Scientific, U.S.A.).

### Parasite and animals

*T. gondii* tachyzoites (RH strain) were provided by Peking University Health Science Center (Beijing, China). Snails confirmed to be infected with *Schistosoma japonicum* cercariae were purchased from the Jiangsu Institute of Parasitic Diseases. *Plasmodium berghei* was passaged in the laboratory. Fish confirmed to be infected with *Clonorchis sinensis* metacercariae were donated by the Department of Parasitology, Sun Yat-Sen University. Six-week-old female BALB/c mice were purchased from Beijing Vital River Laboratory Animal Technology, and feeding in specific pathogens free environment. After collecting blood from the canthus, mice were anesthetized with isoflurane and then sacrificed by cervical dislocation.

### Preparation of test serum

*T. gondii* tachyzoites were cultured in human foreskin fibroblasts (HFFs) [21]. HFFs infected with tachyzoites were collected, centrifuged at 985×*g* for 10 min and washed twice with phosphate-buffered saline (PBS). Each pellet was resuspended in an appropriate amount of PBS, sonicated, and centrifuged at 12000×*g* for 15 min at 4 °C [22]. The supernatant, which contained STAg from *T. gondii*, was collected, aliquoted, and stored at − 80 °C. The concentration of STAg was measured using a BCA protein assay kit.

Forty mice were subcutaneously injected with a mixture of STAg (20 μg per mouse) and an equivalent volume of Freund’s complete adjuvant (Sigma, U.S.A.). Two weeks later, a second immunization was performed with an emulsion of STAg in an equal amount of Freund’s incomplete adjuvant (Sigma, U.S.A.). One week later, a third immunization was performed with the same dose and method as the second immunization. One week after the final immunization, serum was collected and analyzed by ELISA. Negative control serum was prepared from twenty mice immunized with PBS.

Thirty mice randomly divided into 10 mice per group were used to prepare serum samples of *C. sinensis*, *S. japonicum* and *P. berghei* infection. Each mouse was gavaged with 45 metacercariae, and *C. sinensis*-positive serum was collected one month later [23]. *S. japonicum*-positive serum was prepared by infecting mice (40 cercariae per mouse) percutaneously in a shaved region of the abdomen [24]. Mice were intraperitoneally inoculated with 1 × 10^6^ *P. berghei* parasites [25]. Tail vein blood smears were prepared and stained with Giemsa. Serum was collected when the percentage of infected red blood cells exceeded 50%. All serum samples were maintained at − 20 °C for later use.

### Detection of serum by dot-IGSS assay

#### Preparation of a colloidal gold-labeled secondary antibody

Colloidal gold particles (5 nm) were prepared by the tannic acid-trisodium citrate mixed reduction method [26]. Solution A (2.5 mL of 1% HAuCl and 197.5 mL of ddH_2_O) and solution B (10 mL of 1% sodium citrate, 1.75 mL of 1% citric acid, 0.5 mL of 0.1 M K_2_CO_3_, and 37.5 mL of ddH_2_O) were prepared separately and preheated to 60 °C with magnetic stirring. Solution B was quickly poured into solution A, and the mixture was boiled for 5 min after it turned dark red. The pH of the colloidal gold solution was adjusted to 9.0 using 0.1 mol/L K_2_CO_3_. Then, 0.2 mL of goat anti-mouse or goat anti-human IgG (2 mg/mL) (Zhongshan Gold Bridge, China) was added to 40 mL of the above solution, and the mixture was stirred continuously for 20 min. Then, 4 mL of 10% bovine serum albumin (BSA) was added, and the mixture was stirred for 20 min. The supernatant was collected after centrifugation at 1500×*g* for 30 min. The precipitate was collected after centrifugation at 12000×*g* for 60 min and dissolved in 4 mL of Tris Buffered Saline (TBS) (1.12 g of Tris, 8.8 g of NaCl, and 1 L of ddH_2_O, adjust pH to 7.5 with hydrochloric acid). The colloidal gold- labeled secondary antibody was stored at − 20 °C.

### Dot-IGSS assay

Pieces of nitrocellulose (NC) membrane were placed in separate wells of a 96-well plate [16]. rTgPrx (1 mg/mL, 1 μL) was added to the NC membranes, and the membranes were allowed to air dry. At the beginning of the experiment, the type of blocking solution, the blocking time, the dilution ratios of mouse serum and the colloidal gold-labeled secondary antibody were optimized (Additional files 1: Fig. S4, S5). Determined after exploration, the dried NC membranes were blocked in TBS containing 1% BSA and 10% goat serum at 37 °C for 30 min. Diluted serum (mouse, 1:200; human, 1:100) was added to the sample wells and incubated at 37 °C for 1.5 h. The serum was removed from each well, and the wells were washed three times with TBS for 5 min each. Diluted secondary antibody solution (1:20) was added to each well, and the plate was incubated at 37 °C for 1.5 h. The secondary antibody solution was removed from each well, and the plate was washed sequentially with TBS, deionized water and distilled water. Silver nitrate solution was added to each well, and the plate was incubated in the dark for 7 min. The reaction was terminated by rinsing with ionized water, and the membranes were air dried. Brownish gray or brownish yellow spots on the NC membranes indicated positive serum. The negative control serum and blank control were also tested via the above method.

### Assessment of the sensitivity, specificity, and reproducibility of the dot-IGSS assay

Serum samples from mice and humans were simultaneously analyzed by Dot-IGSS with rTgPrx as the antigen (rTgPrx-Dot-IGSS), ELISA using rTgPrx as the antigen (rTgPrx-ELISA), Western blotting and a commercial ELISA kit for sensitivity analysis. Serum samples from mice infected with *C. sinensis*, *S. japonicum* and *P. berghei* were used for specificity analysis. Each serum sample was tested three times for repeatability analysis.

### Diagnosis by ELISA with serum.

Serum samples from mice and humans were analyzed with a commercial ELISA kit for the anti-*Toxoplasma* IgG antibody (Haitai Biotech, China) following the manufacturer’s protocol. In brief, diluted serum (1:100, 100 μL per well) was added to the wells and incubated at 37 °C for 30 min. After washing, an enzyme-labeled antibody (50 μL per well) was added, and the plate was incubated at 37 °C for 30 min. The colored substrate solution was added, and the plate was incubated at 37 °C for 15 min in the dark. The reaction was then stopped, and the absorbance at 450 nm (A450 nm) was measured with a microplate reader (ASYS-Hitech GmbH, U.S.A.).

Serum samples from mice and humans were analyzed by rTgPrx-ELISA. A 96-well microtiter plate was coated with rTgPrx (1 μg/well) and incubated at 4 °C for 12 h. After blocking at 37 °C for 1 h, diluted serum (mouse, 1:200; human, 1:100) was added to the wells, and the plate was incubated at 37 °C for 12 h. Unbound serum antibody was then washed away, and the samples were incubated with horseradish peroxidase (HRP)-conjugated goat anti-mouse IgG (diluted 1:5000) or goat anti-human IgG (diluted 1:2500) at 37 °C for 2 h. O-phenylenediamine was the substrate solution; after it was added, the plate was protected from light for 15 min. The reaction was stopped, and the absorbance at 492 nm (A492 nm) was measured within 20 min. Each sample was independently analyzed three times, and the average value was calculated [27].

### Western blotting

rTgPrx (20 μg) was separated via sodium dodecyl sulfate-polyacrylamide gel electrophoresis (SDS-PAGE) on 12% gels and transferred to polyvinylidene difluoride membranes. Membranes were incubated with mouse serum or human pregnancy serum (diluted 1:100) at 4 °C overnight and were then probed with an HRP-conjugated goat anti-mouse or anti-human IgG antibody (diluted 1:5000, Zhongshan Gold Bridge, China).

### Statistical analysis

Data were analyzed using SPSS software. The positive rates were compared between groups with corrected chi-square tests or Fisher’s exact test. Fisher’s exact test was used to compare the positive rate of different batches. A significance level (α) of 0.05 was selected, and *P* < 0.05 was considered to indicate statistical significance.

## Results

### Identification of the rTgPrx

**Fig. 1:**
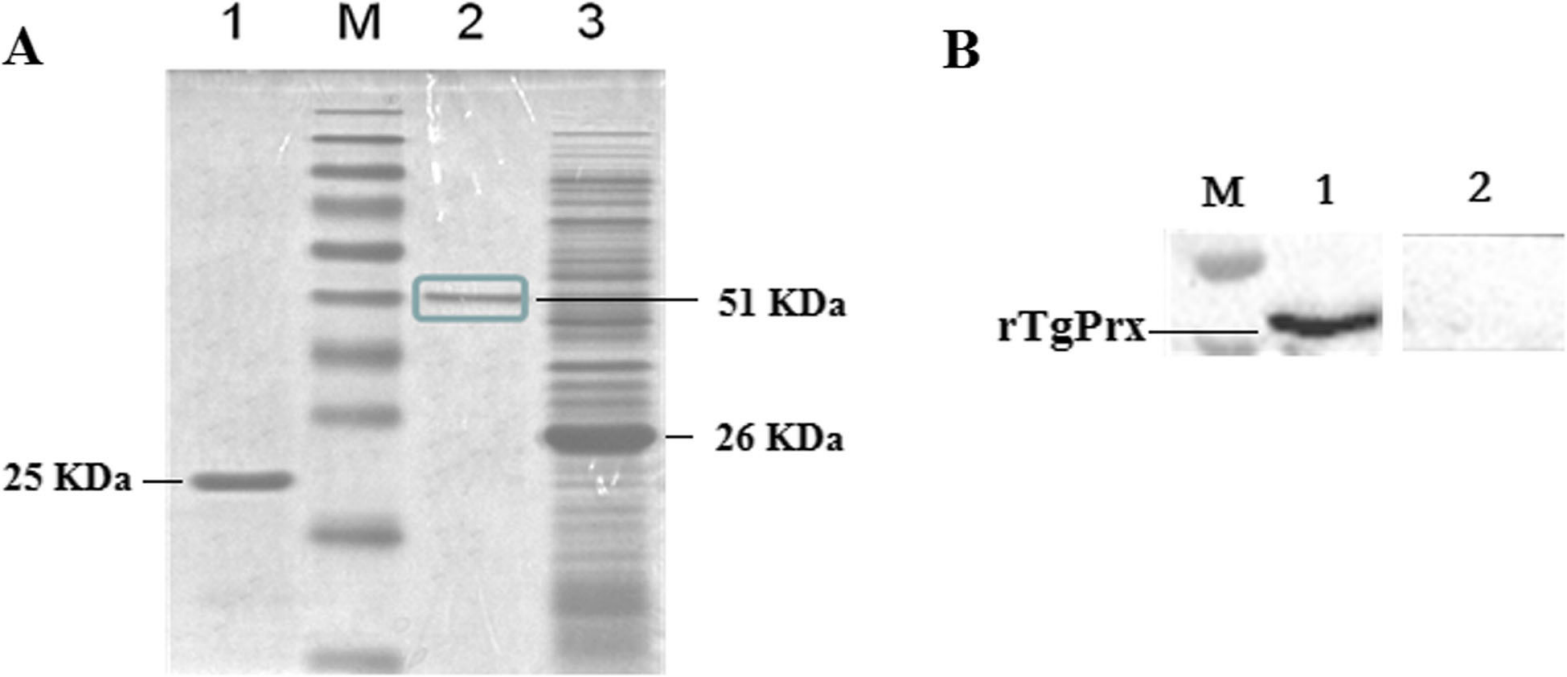
Detection of rTgPrx by SDS-PAGE and Western blotting. **a**: rTgPrx was expressed in *E. coli*, purified using GST affinity chromatography, analyzed by SDS-PAGE on 12% gels, and stained with Coomassie brilliant blue. M: marker. Lane 1: The molecular weight of rTgPrx digested by PreScission Protease was 25 kDa. Lane 2: GST-tagged rTgPrx was purified by GST affinity chromatography and had a molecular weight of 51 kDa. Lane 3: SDS-PAGE showed that unpurified rTgPrx was expressed in *E. coli.* **b**: The immunoreactivity of rTgPrx was detected by Western blotting. M: marker. Lane 1: rTgPrx was reacted with rabbit anti-*T.gondii* serum. Lane 2: rTgPrx was reacted with negative serum

Expression of the positive recombinant plasmid pGEX-6P-1/TgPrx in *E. coli* was optimized from IPTG concentration, induced temperature and times (Additional files 1: Fig. S1, S2, S3). Finally, pGEX-6P-1/TgPrx was induced by 0.1mM IPTG at 25 °C for 12 h, and the protein was purified by GST affinity chromatography. SDS-PAGE showed that the molecular weight of GST-tagged rTgPrx was 51 kDa (Fig. 1a). The GST tag was cleaved from rTgPrx with PreScission Protease. The molecular weights of the GST tag and rTgPrx were 26 kDa and 25 kDa, respectively (Fig. 1a). The analysis of Imag J software showed that the purity of rTgPrx protein on SDS-PAGE was calculated as 95.6%. The purifiedrTgPrx could be recognized by rabbit anti-*T.gondii* serum. The result of Western blotting showed the specific strips (Fig. 1b). Those results confirmed the acquisition of purified rTgPrx.

### Evaluation of *T. gondii* infection in mice by dot-IGSS

#### Testing of mouse serum samples

The diagnostic potential of rTgPrx was evaluated by Dot-IGSS. Serum positive for *T. gondii* was collected from mice immunized with STAg (the titers of immunized mouse serum were 1:800 and 1:1600), while serum negative for *T. gondii* was obtained from mice immunized with PBS. As determined by checkerboard titration, the optimal dilution ratios of the mouse serum and goat anti-mouse gold-labeled antibody for the Dot-IGSS assay were 1:200 and 1:20, respectively (Additional files 1: Fig. S5). In this study, 40 positive and 20 negative serum samples from mice were analyzed by Dot-IGSS using rTgPrx as the antigen (Additional files 1: Fig. S6). Thirty-nine of the 40 serum samples from mice immunized with STAg were positive, while all of the 20 serum samples from mice immunized with PBS were negative.

### Sensitivity analysis

Positive serum samples from mice immunized with STAg were simultaneously analyzed by rTgPrx-Dot-IGSS, rTgPrx-ELISA, Western blotting (Additional files 1: Fig. S7), and a commercial ELISA kit for the anti-*Toxoplasma* IgG antibody. The positive *T. gondii* infection rates among the 40 serum samples were 97.5% (39/40) for rTgPrx-Dot-IGSS, 95% (38/40) for rTgPrx-ELISA, 90% (36/40) for Western blotting, and 100% (40/40) for the commercial ELISA kit (Table 1). The positive rates obtained with rTgPrx-Dot-IGSS, rTgPrx-ELISA and Western blotting did not differ significantly from those obtained with the commercial ELISA kit (*P* = 1.000, *P* = 0.494, and *P* = 0.116, respectively), indicating that the sensitivity of rTgPrx-Dot-IGSS is comparable to that of the commercial ELISA kit.

**Table 1:** Sensitivity analysis of rTgPrx-Dot-IGSS

Test method	Number of samples^a^	Number of positive samples	Positive rate
rTgPrx-Dot-IGSS	40	39	97.5%
rTgPrx-ELISA	40	38	95.0%
Western blotting	40	36	90.0%
Commercial ELISA kit	40	40	100%

Note: ^a^ Serum from mice immunized with STAg. The serum titers were 1:800 and 1:1600, as determined by ELISA

### Specificity analysis

Serum was collected from mice infected with PBS, *C. sinensis*, *S. japonicum* or *P. berghei* and analyzed by rTgPrx-Dot-IGSS, rTgPrx-ELISA, Western blotting and a commercial ELISA kit for the anti-*Toxoplasma* IgG antibody. The results of Dot-IGSS, ELISA and Western blotting using rTgPrx as the antigen showed that all serum samples were negative and lacked cross-reactivity. However, one serum sample from a mouse infected with *S. japonicum* was determined to be positive by the commercial ELISA kit. These results indicate that rTgPrx-Dot-IGSS has higher specificity than this commercial ELISA kit (Table 2).

**Table 2:** Specificity analysis of rTgPrx-Dot-IGSS

Group	Number of samples	rTgPrx-Dot-IGSS		rTgPrx-ELISA		Western blotting		Commercial ELISA kit	
		Positive	Negative	Positive	Negative	Positive	Negative	Positive	Negative
PBS	20	0	20	0	20	0	20	0	20
*Clonorchis sinensis*	10	0	10	0	10	0	10	0	10
*Schistosoma japonicum*	10	0	10	0	10	0	10	1	9
*Plasmodium berghei*	10	0	10	0	10	0	10	0	10

### Repeatability analysis

Forty serum samples positive for *T. gondii* and 20 serum samples negative for *T. gondii* from mice were analyzed in triplicate by rTgPrx-Dot-IGSS. The results of the repeated tests were consistent(*P <* 0.0001), showing that the rTgPrx-Dot-IGSS assay has good repeatability and stability.

### Detection of *T. gondii* infection in pregnancy serum by rTgPrx-dot-IGSS

A total of 540 pregnancy serum samples were collected and analyzed with a commercial ELISA kit for the anti-*Toxoplasma* IgG antibody. Eighty-three of the serum samples were positive; thus, the *T. gondii* infection rate among the pregnant women was 15.4%. The 83 serum samples confirmed to be positive with the commercial ELISA kit were analyzed by rTgPrx-Dot-IGSS, rTgPrx-ELISA and Western blotting. The positive rates were 95.18% (79/83) for rTgPrx-Dot-IGSS, 92.77% (77/83) for rTgPrx-ELISA, and 86.75% (72/83) for Western blotting (Table 3). The positive rate obtained with rTgPrx-Dot-IGSS did not differ significantly from that obtained with the commercial ELISA kit (*P* = 0.120), while those determined by rTgPrx-ELISA and Western blotting were appreciably lower (*P* = 0.028 and *P* = 0.001, respectively). These results indicate that rTgPrx-Dot-IGSS is comparable to the commercial ELISA kit for the diagnosis of *T. gondii* infection.

**Table 3:** Detection of *Toxoplasma* in pregnancy serum samples

Test method	Commercial ELISA kit		Test result		
	Positive	Negative	Positive	Negative	Positive rate^a^
rTgPrx-Dot-IGSS	83	60	79	64	95.18%
rTgPrx-ELISA	83	60	77	66	92.77%
Western blotting	83	30	72	41	86.75%

Note: ^a^ The positive rate is the number of positive cases determined with rTgPrx-Dot-IGSS, rTgPrx-ELISA or Western blotting divided by the number of positive cases determined with the commercial ELISA kit × 100%

## Discussion

As molecular biology techniques have been developed, recombinant antigens have recently been used instead of natural antigens, such as surface antigens (SAG1), dense granular antigens (GRA1 and GRA7) and rhoptry antigens (ROP18), for diagnostic tests [28–31]. Mass and standardized production of recombinant antigens is straightforward [9]. In this study, we constructed the recombinant plasmid pGEX-6P-1-TgPrx. pGEX-6P-1 is a highly efficient expression vector containing a GST tag, making recombinant protein purification easy and efficient [32]. pGEX-6P-1-TgPrx was transformed into *E. coli* BL21 for expression. We optimized the experimental conditions to maximize the expression levels of soluble proteins by using a low concentration of IPTG, reducing the induction temperature and extending the induction time. The results of preliminary experiments showed that the presence of the GST tag could affect the specificity of rTgPrx and cause nonspecific reactions during the detection of serum antibodies. Therefore, the GST tag was cleaved with PreScission Protease. PreScission Protease is a human rhinovirus type 3C protease containing a GST tag that enables fixation of proteins and removal of the GST tag [33]. The digestion time and volume of PreScission Protease used were adjusted according to the concentration of rTgPrx. The analysis of Imag J software confirmed that 95.6% purity of rTgPrx protein was obtained as the detection antigen. The immunoreactivity of purified rTgPrx was further verified by Western blotting.

In recent years, dot-based immunoassay technology (Dot-Blot, Dot-ELISA and dot immunogold filtration assay, et al) has attracted the attention of scholars [34–36]. Initially, we used rTgPrx as the diagnostic antigen to establish a Dot-IGSS method for the detection of *T. gondii* infection in mice. The optimal dilution ratios of mouse serum and the colloidal gold-labeled secondary antibody, the optimal blocking solution, and the optimal blocking time for this method were explored. The optimal dilution ratios of mouse serum and goat anti-mouse IgG were determined to be 1:200 and 1:20, respectively. TBS containing 1% BSA and 10% sheep serum was used as the blocking solution, and incubation was performed for 30 min at 37 °C. rTgPrx-Dot-IGSS was used to analyze 40 serum samples from mice immunized with STAg. The results indicated a positive rate of 97.5% and no cross-reactivity. A previous study showed that the sensitivity and specificity of IgG ELISA with a single recombinant surface antigen (SAG1) or recombinant dense granular antigens (GRA1 and GRA7) in captive jaguars were 92.5 ~ 97.5% and 83.3 ~ 91.6%, respectively, and that the sensitivity and specificity were significantly increased when these antigens were mixed [30]. Therefore, the sensitivity of rTgPrx-Dot-IGSS could be increased by mixing rTgPrx with other specific antigens.

Furtherly, rTgPrx-Dot-IGSS was used to detect human Toxoplasmosis. Eighty-three positive serum samples were identified among 540 pregnancy serum samples by screening with a commercial ELISA kit for the anti-*Toxoplasma* IgG antibody. The 83 positive serum samples and 60 negative samples were reanalyzed by rTgPrx-Dot-IGSS, rTgPrx-ELISA and Western blotting. These results showed that rTgPrx-Dot-IGSS could be used to diagnose toxoplasmosis. However, 4 samples identified as positive by the commercial ELISA kit were identified as negative with rTgPrx-Dot-IGSS. This discrepancy has a few possible explanations. First, the antigen coating in the commercial ELISA kit was a mixed antigen with whole tachyzoite lysate, while rTgPrx is a single purified antigen with high specificity and slightly lower sensitivity than the antigen in the ELISA kit. Purification and mixing of highly specific antigens should be considered for future diagnosis. Second, the commercial ELISA kit might have produced false positive results due to antigen impurity, contamination of the detection reagents or experimental errors. Therefore, the specificity and sensitivity of commercial ELISA kits from different companies should be compared.

In addition, Only detection of IgG in serum was shown in the results. As we known, IgM appeared firstly during infection, but it didn’t last long. In the pre-experimental stage, we detected IgM from partly immunized mice. The results showed that rTgPrx-Dot-IGSS was feasible. However, IgM of the pregnancy serum hadn’t been tested.

## Conclusions

The rTgPrx could be described as valuable diagnostic antigens of toxoplasmosis. In this study, the rTgPrx-Dot-IGSS assay was established by detecting *T.gondii* infection in mice, and initially applied to the detection of human toxoplasmosis. It exhibited not only simple operation, a low cost and intuitive results but also moderate sensitivity, good specificity and strong repeatability. Thus, this method is a promising diagnostic tool for clinical diagnosis of toxoplasmosis.

## Supplementary information

**Additional file 1: Fig. S1.** SDS-PAGE analysis of pGEX-6P-1/TgPrx/BL21 expression products induced by different concentrations of IPTG. **Fig. S2.** SDS-PAGE analysis of pGEX-6P-1/TgPrx/BL21 expression products at different induction times. **Fig. S3.** SDS-PAGE analysis of optimized expression temperature of pGEX-6P-1/TgPrx/BL21. **Fig. S4.** The optimization of the type of blocking solution and the blocking time for Dot-IGSS assay. **Fig. S5.** The antibodies dilution for Dot-IGSS assay determined by checkerboard titration. **Fig. S6.** Detection of *T. gondii* infection in mice by rTgPrx-Dot-IGSS. **Fig. S7.** Detection of *T. gondii* infection in mice by Western blotting.

## Data Availability

The datasets included in the present study are available from the corresponding author on reasonable request.

## Abbreviations

rTgPrx: Recombinant *T. gondii* peroxiredoxin protein
Dot-IGSS: Dot-immunogold-silver staining
ELISA: Enzyme-linked immunosorbent assay
IgG: Immunoglobulin G
*T. gondii*: *Toxoplasma gondii*
