# Supplementary material for: Evaluation of toxoplasmosis in pregnant women using dot-immunogold-silver staining with recombinant Toxoplasma gondii peroxiredoxin protein
Source: BMC Infect Dis. 2020 Sep 22;20:694. doi: 10.1186/s12879-020-05414-8 (PMC7507715; doi:10.1186/s12879-020-05414-8)
Supplement: Supplementary file 1 — Additional file 1: Fig. S1. SDS-PAGE analysis of pGEX-6P-1/TgPrx/BL21 expression products induced by different concentrations of IPTG. Fig. S2. SDS-PAGE analysis of pGEX-6P-1/TgPrx/BL21 expression products at different induction times. Fig. S3. SDS-PAGE analysis of optimized expression temperature of pGEX-6P-1/TgPrx/BL21. Fig. S4. The optimization of the type of blocking solution and the blocking time for Dot-IGSS assay. Fig. S5. The antibodies dilution for Dot-IGSS assay determined by checkerboard titration. Fig. S6. Detection of T. gondii infection in mice by rTgPrx-Dot-IGSS. Fig. S7. Detection of T. gondii infection in mice by Western blotting. [file 12879_2020_5414_MOESM1_ESM.zip › 12879_2020_5414_MOESM1_ESM/Additional files-2020.9.18_ESM.docx]

**Fig S1. SDS-PAGE analysis of pGEX-6P-1/TgPrx/BL21 expression products induced by different concentrations of IPTG**

1:0.5mM IPTG. 2: 0.4mM IPTG. 3:0.3mM IPTG. 4: 0.2mM IPTG.

5:0.1mM IPTG. M: marker

**Fig S2. SDS-PAGE analysis of pGEX-6P-1/TgPrx/BL21 expression products at different induction times**

1: E.coli BL21 induced expression for 4 hours.

2: pGEX-6P-1/BL21 induced expression for 4 hours.

3: recombinant plasmid expression was not induced.

4: recombinant plasmid induced expression for 12 hours.

5: recombinant plasmid induced expression for 10 hours.

6: the supernatant of recombinant plasmid induced expression for 8h.

7: the supernatant of recombinant plasmid induced expression of 6h.

8: the supernatant of recombinant plasmid induced expression of 4h.

9: the supernatant of recombinant plasmid induced expression of 2h.

M: marker.

**Fig S3. SDS-PAGE analysis of optimized expression temperature of pGEX-6P-1/TgPrx/BL21**

1: Total protein induced by pGEX-6P-1/TgPrx/BL21 at 37℃.

2: Total protein induced by pGEX-6P-1/TgPrx/BL21 at 25℃.

3: Total protein induced by pGEX-6P-1/TgPrx/BL21 at 16°C.

4: Total protein induced by pGEX-6P-1/BL2(control);

5: Total protein induced by BL21 (control).

**Fig S4. The** **optimization of** **the type of blocking solution and the blocking time for Dot-IGSS assay.**

A: TBS. B: 1%BSA+TBS. C: 1%BSA+TBS+10%FBS.

D: 1%BSA+TBS+10% goat serum.

Ab(+): Mouse serum from *T.gondii* infection. Ab(-): Negative mouse serum.

**Fig S5. The antibodies dilution for Dot-IGSS assay determined by checkerboard titration.**

Ab1(+): Mouse serum from *T.gondii* infection. Ab1(-): Negative mouse serum.

1～11: Mouse serum from *T.gondii* or PBS infection were diluted by 1/10, 1/50, 1/100, 1/200, 1/400, 1/800, 1/1600, 1/3200, 1/6400, 1/12800 and 1/25600.

Ab2：Goat anti-mouse gold-labeled antibody(1:5～1:80).

**Fig S6.** **Detection of *T. gondii* infection in mice by rTgPrx-Dot-IGSS**

Mouse serum from *T.gondii* infection or PBS were detected by rTgPrx-Dot-IGSS. The positive serum presented brownish yellow spots on the NC membranes, while the negative serum had no spots.

**Fig S7.** **Detection of *T. gondii* infection in mice by Western blotting**

Mouse serum from *T.gondii* infection or PBS were detected by Western blotting with rTgPrx as antigen. The positive serum presented obvious strips with 25kDa, while the negative serum had no strips. M: marker. Lane 1～4: positive serum. Lane 5～8: negative serum.
