# Supplementary figures and images for: Evaluation of toxoplasmosis in pregnant women using dot-immunogold-silver staining with recombinant Toxoplasma gondii peroxiredoxin protein
Source: BMC Infect Dis. 2020 Sep 22;20:694. doi: 10.1186/s12879-020-05414-8 (PMC7507715; doi:10.1186/s12879-020-05414-8)

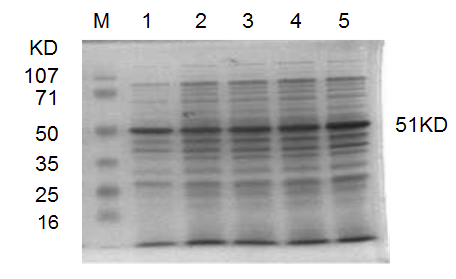

Supplement: Supplementary file 1 — Additional file 1: Fig. S1. SDS-PAGE analysis of pGEX-6P-1/TgPrx/BL21 expression products induced by different concentrations of IPTG. Fig. S2. SDS-PAGE analysis of pGEX-6P-1/TgPrx/BL21 expression products at different induction times. Fig. S3. SDS-PAGE analysis of optimized expression temperature of pGEX-6P-1/TgPrx/BL21. Fig. S4. The optimization of the type of blocking solution and the blocking time for Dot-IGSS assay. Fig. S5. The antibodies dilution for Dot-IGSS assay determined by checkerboard titration. Fig. S6. Detection of T. gondii infection in mice by rTgPrx-Dot-IGSS. Fig. S7. Detection of T. gondii infection in mice by Western blotting. [file 12879_2020_5414_MOESM1_ESM.zip › 12879_2020_5414_MOESM1_ESM/Fig S1R3.tif]

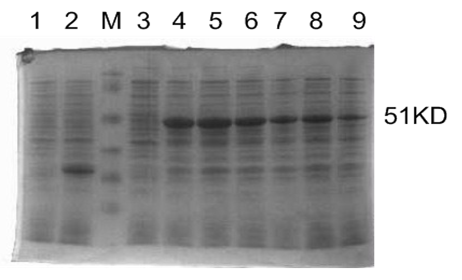

Supplement: Supplementary file 1 — Additional file 1: Fig. S1. SDS-PAGE analysis of pGEX-6P-1/TgPrx/BL21 expression products induced by different concentrations of IPTG. Fig. S2. SDS-PAGE analysis of pGEX-6P-1/TgPrx/BL21 expression products at different induction times. Fig. S3. SDS-PAGE analysis of optimized expression temperature of pGEX-6P-1/TgPrx/BL21. Fig. S4. The optimization of the type of blocking solution and the blocking time for Dot-IGSS assay. Fig. S5. The antibodies dilution for Dot-IGSS assay determined by checkerboard titration. Fig. S6. Detection of T. gondii infection in mice by rTgPrx-Dot-IGSS. Fig. S7. Detection of T. gondii infection in mice by Western blotting. [file 12879_2020_5414_MOESM1_ESM.zip › 12879_2020_5414_MOESM1_ESM/Fig S2R3.tif]

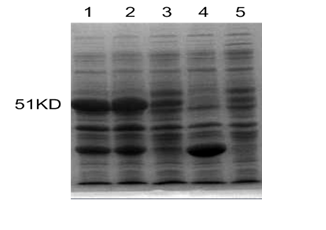

Supplement: Supplementary file 1 — Additional file 1: Fig. S1. SDS-PAGE analysis of pGEX-6P-1/TgPrx/BL21 expression products induced by different concentrations of IPTG. Fig. S2. SDS-PAGE analysis of pGEX-6P-1/TgPrx/BL21 expression products at different induction times. Fig. S3. SDS-PAGE analysis of optimized expression temperature of pGEX-6P-1/TgPrx/BL21. Fig. S4. The optimization of the type of blocking solution and the blocking time for Dot-IGSS assay. Fig. S5. The antibodies dilution for Dot-IGSS assay determined by checkerboard titration. Fig. S6. Detection of T. gondii infection in mice by rTgPrx-Dot-IGSS. Fig. S7. Detection of T. gondii infection in mice by Western blotting. [file 12879_2020_5414_MOESM1_ESM.zip › 12879_2020_5414_MOESM1_ESM/Fig S3R3.tif]

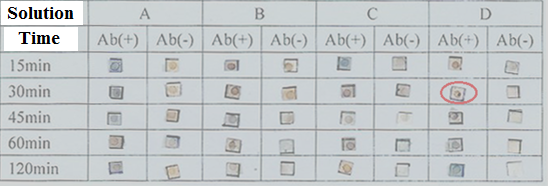

Supplement: Supplementary file 1 — Additional file 1: Fig. S1. SDS-PAGE analysis of pGEX-6P-1/TgPrx/BL21 expression products induced by different concentrations of IPTG. Fig. S2. SDS-PAGE analysis of pGEX-6P-1/TgPrx/BL21 expression products at different induction times. Fig. S3. SDS-PAGE analysis of optimized expression temperature of pGEX-6P-1/TgPrx/BL21. Fig. S4. The optimization of the type of blocking solution and the blocking time for Dot-IGSS assay. Fig. S5. The antibodies dilution for Dot-IGSS assay determined by checkerboard titration. Fig. S6. Detection of T. gondii infection in mice by rTgPrx-Dot-IGSS. Fig. S7. Detection of T. gondii infection in mice by Western blotting. [file 12879_2020_5414_MOESM1_ESM.zip › 12879_2020_5414_MOESM1_ESM/Fig S4R3.tif]

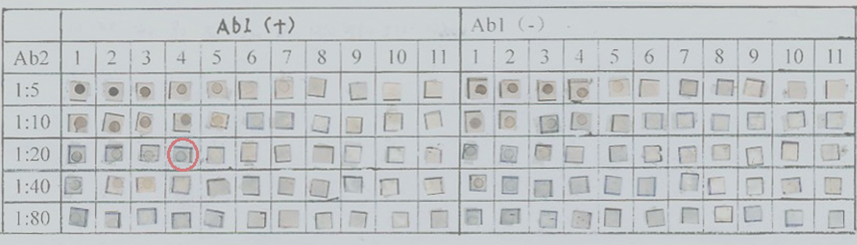

Supplement: Supplementary file 1 — Additional file 1: Fig. S1. SDS-PAGE analysis of pGEX-6P-1/TgPrx/BL21 expression products induced by different concentrations of IPTG. Fig. S2. SDS-PAGE analysis of pGEX-6P-1/TgPrx/BL21 expression products at different induction times. Fig. S3. SDS-PAGE analysis of optimized expression temperature of pGEX-6P-1/TgPrx/BL21. Fig. S4. The optimization of the type of blocking solution and the blocking time for Dot-IGSS assay. Fig. S5. The antibodies dilution for Dot-IGSS assay determined by checkerboard titration. Fig. S6. Detection of T. gondii infection in mice by rTgPrx-Dot-IGSS. Fig. S7. Detection of T. gondii infection in mice by Western blotting. [file 12879_2020_5414_MOESM1_ESM.zip › 12879_2020_5414_MOESM1_ESM/fig S5R3.tif]

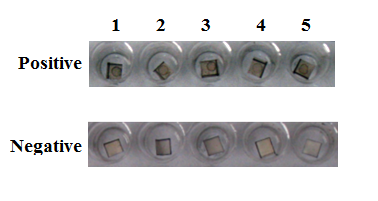

Supplement: Supplementary file 1 — Additional file 1: Fig. S1. SDS-PAGE analysis of pGEX-6P-1/TgPrx/BL21 expression products induced by different concentrations of IPTG. Fig. S2. SDS-PAGE analysis of pGEX-6P-1/TgPrx/BL21 expression products at different induction times. Fig. S3. SDS-PAGE analysis of optimized expression temperature of pGEX-6P-1/TgPrx/BL21. Fig. S4. The optimization of the type of blocking solution and the blocking time for Dot-IGSS assay. Fig. S5. The antibodies dilution for Dot-IGSS assay determined by checkerboard titration. Fig. S6. Detection of T. gondii infection in mice by rTgPrx-Dot-IGSS. Fig. S7. Detection of T. gondii infection in mice by Western blotting. [file 12879_2020_5414_MOESM1_ESM.zip › 12879_2020_5414_MOESM1_ESM/Fig S6R3.tif]

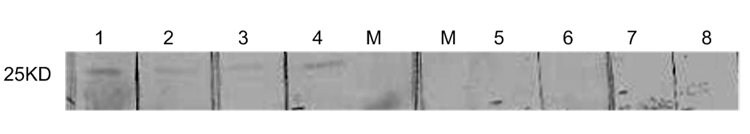

Supplement: Supplementary file 1 — Additional file 1: Fig. S1. SDS-PAGE analysis of pGEX-6P-1/TgPrx/BL21 expression products induced by different concentrations of IPTG. Fig. S2. SDS-PAGE analysis of pGEX-6P-1/TgPrx/BL21 expression products at different induction times. Fig. S3. SDS-PAGE analysis of optimized expression temperature of pGEX-6P-1/TgPrx/BL21. Fig. S4. The optimization of the type of blocking solution and the blocking time for Dot-IGSS assay. Fig. S5. The antibodies dilution for Dot-IGSS assay determined by checkerboard titration. Fig. S6. Detection of T. gondii infection in mice by rTgPrx-Dot-IGSS. Fig. S7. Detection of T. gondii infection in mice by Western blotting. [file 12879_2020_5414_MOESM1_ESM.zip › 12879_2020_5414_MOESM1_ESM/Fig S7R3.tif]
